# Ischemic Type of Central Vein Occlusion in a Patient With Bietti Crystalline Dystrophy: A Longitudinal Follow-Up of 12 Years

**DOI:** 10.7759/cureus.107116

**Published:** 2026-04-15

**Authors:** Hamit Ali, Mehmet Kocabey, Ziya Ayhan, Ahmet Okay Caglayan, Ali Osman Saatci

**Affiliations:** 1 Department of Ophthalmology, Dokuz Eylül University, Izmir, TUR; 2 Department of Medical Genetics, Dokuz Eylül University, Izmir, TUR

**Keywords:** bietti crystalline dystrophy, retinal neovascularization, retinal vein occlusion, retinitis pigmentosa, rvo

## Abstract

Bietti crystalline dystrophy (BCD) is a rare chorioretinal dystrophy associated with mutations in the CYP4V2 gene, characterized by intraretinal crystalline deposits and progressive chorioretinal degeneration. Retinal vein occlusion (RVO) is exceedingly rare in BCD, with only one previously reported case of tributary RVO without a detailed clinical description. We hereby describe a 44-year-old man with genetically proven stage 2 BCD who developed right central RVO (CRVO) eight years following the initial diagnosis in 2013. Though he did not notice any new visual disturbance in the right eye, routine fundus examination revealed subtle peripheral microvascular abnormalities and intraretinal small hemorrhages 360 degrees in 2021, and fluorescein angiography demonstrated nearly 360-degree peripheric nonperfusion with scattered microaneurysms, consistent with the diagnosis of ischemic CRVO. Four years later, the patient developed peripheric retinal neovascularizations and a small preretinal hemorrhage in his right eye that was managed solely with scatter laser photocoagulation. Unlike a previously reported case with retinitis pigmentosa and CRVO, in which neovascular complications did not arise, most likely due to extensive peripheral chorioretinal atrophy related to low VEGF levels, our case exhibited less peripheral chorioretinal atrophy; therefore, peripheral ischemia led to the development of retinal neovascularizations. Patients with BCD may not perceive peripheral retinal alterations due to their already compromised central vision; therefore, regular clinical visits seem beneficial to detect potential fundus changes promptly, as demonstrated in the present case.

## Introduction

Bietti crystalline dystrophy (BCD) is a rare autosomal recessive chorioretinal dystrophy initially described by Bietti in 1937 [1]. It is linked to mutations in the CYP4V2 gene, which encodes a cytochrome P450 enzyme involved in fatty acid metabolism and thought to play an important role in lipid metabolism of the retina and RPE cells [2]. BCD is characterized by yellow-white lipid deposits within the ocular structures, notably in the cornea, retina, and choroidal tissues, secondary to abnormal lipid metabolism [3]. As the disease advances, retinal regions exhibiting glistening crystalline deposits, predominantly located at the posterior pole, are replaced by widespread chorioretinal atrophy [4]. Thus, atrophy of the retinal pigment epithelium (RPE), in conjunction with choroidal sclerosis, leads to a progressive visual impairment [5]. Retinal vein occlusion (RVO) may present mostly as branch or central RVO (CRVO), and usually is characterized by a sudden onset of painless vision loss. The most prevalent risk factors include age, hypertension, hyperlipidemia, diabetes mellitus, and glaucoma, and RVO may lead to macular edema, macular ischemia, retinal and/or disc neovascularization, and, in some instances, neovascular glaucoma [6]. RVO is an exceedingly rare occurrence in patients with BCD and other retinochoroidal dystrophies. In a cohort of twelve BCD patients observed over a period ranging from one to five years, Zhang et al. [7] documented a case involving a 60-year-old woman diagnosed with stage 2 disease according to the Yuzawa classification [8]. The patient was diagnosed with an occlusion of a unilateral tributary branch vein and treated with a sector laser photocoagulation. However, the report did not include a detailed case description. Herein, we present the clinical course of a 44-year-old male BCD patient who was diagnosed with a unilateral CRVO during a routine visit in 2021, eight years subsequent to our initial examination in 2013.

## Case presentation

**Figure 1:**
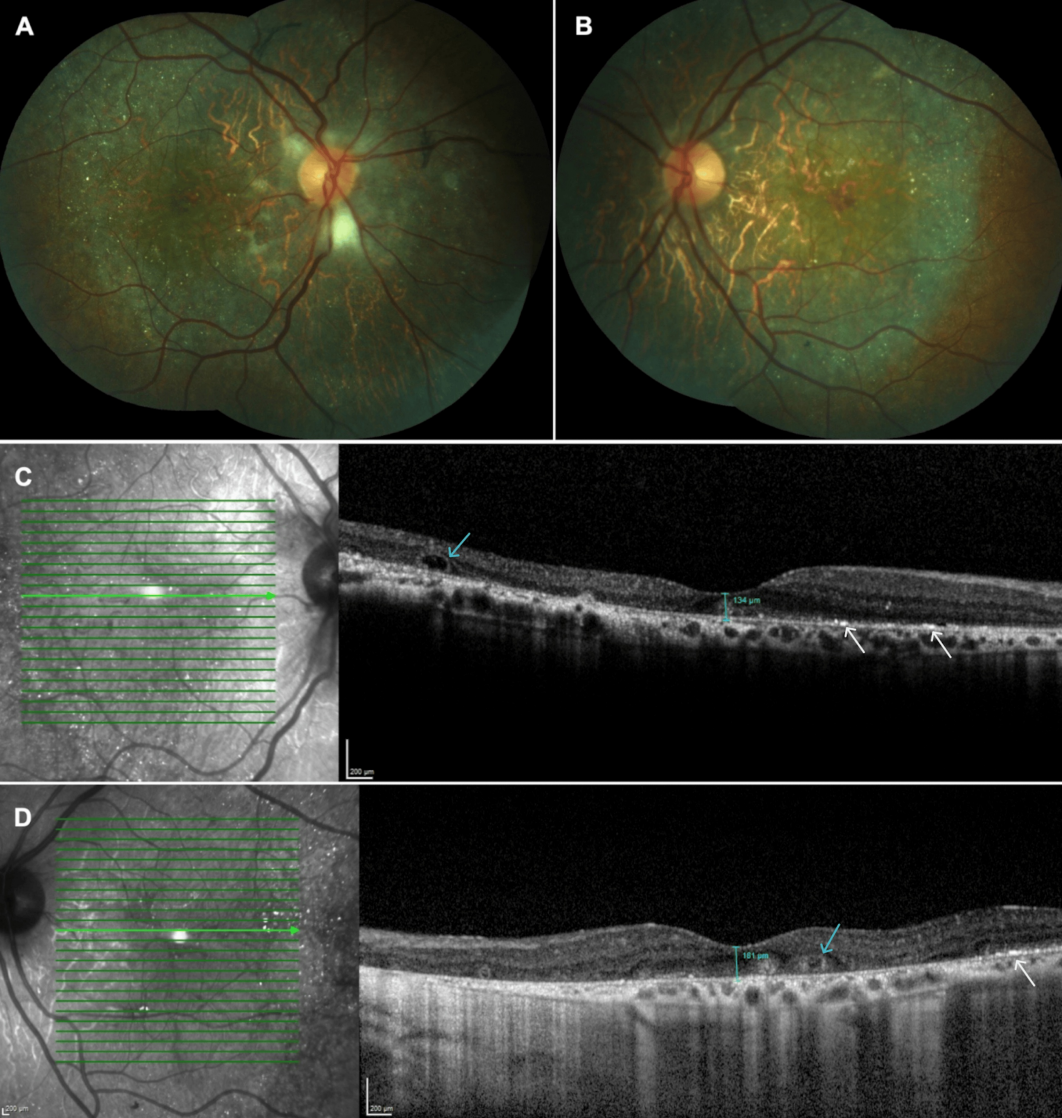
Right and left eyes, 2013 Composite color fundus pictures showing the glistening retinal crystalline deposits with chorioretinal atrophy at the posterior pole (right eye, A, and left eye, B). Spectral-domain optical coherence tomographic sections reveal a few hyperreflective deposits in the sensory retina (white arrows), outer retinal tubulations (blue arrows), and a thinned fovea (right eye, C, and left eye, D).

A 44-year-old man was examined by us in 2013, presenting with a gradual decline in vision in both eyes over the preceding 10 years. His medical and family histories were unremarkable. On ophthalmologic examination, the best-corrected visual acuity was 20/200 in the right eye and 20/100 on the left. The intraocular pressure was within normal limits bilaterally. Anterior segment examination revealed a normal-appearing cornea in both eyes. Fundus examination revealed the presence of bilateral intraretinal crystalline deposits, predominantly situated at the posterior pole, along with chorioretinal atrophy in both eyes (Figures 1A-1B). Spectral-domain optical coherence tomography (OCT) delineated widespread hyperreflective deposits within the RPE-Bruch's membrane complex in both eyes, corresponding to the crystals (Figures 1C-1D). He was diagnosed with stage 2 BCD according to the Yuzawa classification and was recommended to undergo annual examinations [8].

**Figure 2:**
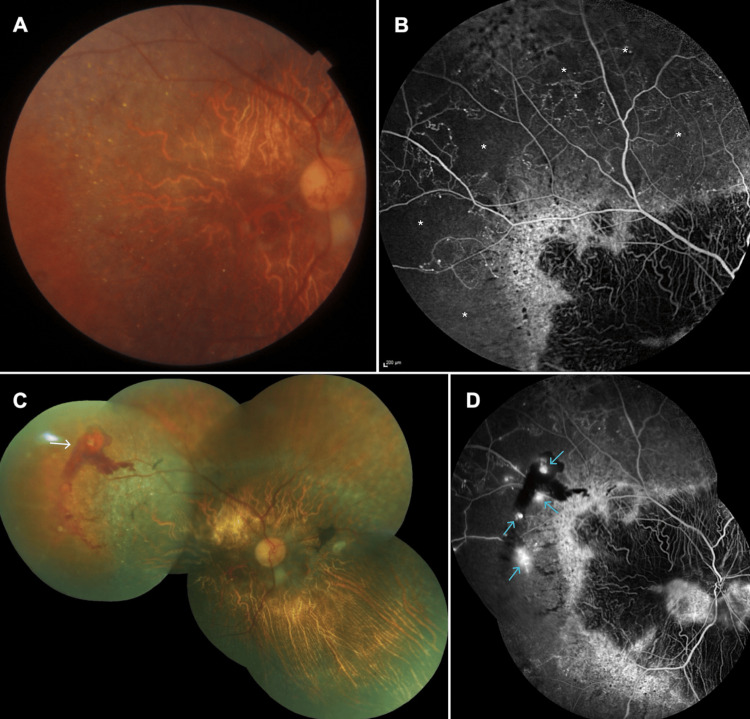
Right eye, 2021 and 2025 2021: Color fundus picture (A) and venous phase of fluorescein angiogram (B) showing the peripheral microvascular changes with capillary nonperfusion areas (asterisks) beyond the central chorioretinal atrophic area. 2025: Composite color fundus picture demonstrating the retinal neovascularization-related preretinal hemorrhage at the temporal periphery (white arrow) (C). The venous phase of the fluorescein angiogram reveals the neovascularizations elsewhere (blue arrows) and the masking effect of the associated hemorrhage (D).

During a routine examination in 2021, the patient did not report any new symptoms, and his visual acuity was 20/200 in OD and 20/200 in OS. Chorioretinal atrophy had progressed slightly, and the number of crystals looked reduced in both eyes. However, there were microvascular alterations and small, scattered intraretinal hemorrhages 360 degrees at the periphery of the right fundus (Triton, Topcon Inc., Oakland, NJ) (Figure 2A). Fluorescein angiogram of the right eye (Heidelberg Retinal Angiography 2) demonstrated nearly 360-degree peripheral nonperfused regions, accompanied by scattered microaneurysms (Figure 2B). We reached a diagnosis of coexistent right CRVO based on the clinical and angiographic findings. As there was no macular edema, we did not employ any anti-VEGF agent. Cardiology and hematology consultations were carried out. No carotid artery stenosis was observed. The comprehensive hematological evaluation yielded negative results for hypercoagulability. The lipid profile was found to be abnormal, with a triglyceride level of 361 mg/dL, low-density lipoprotein cholesterol of 150 mg/dL, and a total cholesterol of 276 mg/dL. Dyslipidemia and atherosclerosis might be the likely underlying triggers in the development of CRVO. Consequently, he was prescribed antiplatelet, antilipid, and antihypertensive medications. A routine eye examination conducted in 2025, without any additional visual complaints, revealed a linear, small preretinal hemorrhage at the superotemporal quadrant of the right eye, indicating the presence of retinal neovascularization (Figure 2C). The fluorescein angiogram demonstrated retinal neovascularization-related leakage in addition to already present nonperfused peripheral areas and masking of preretinal hemorrhage (Figure 2D). Ischemic regions were treated with scattered laser photocoagulation without any intravitreal anti-VEGF administration. 

**Figure 3:**
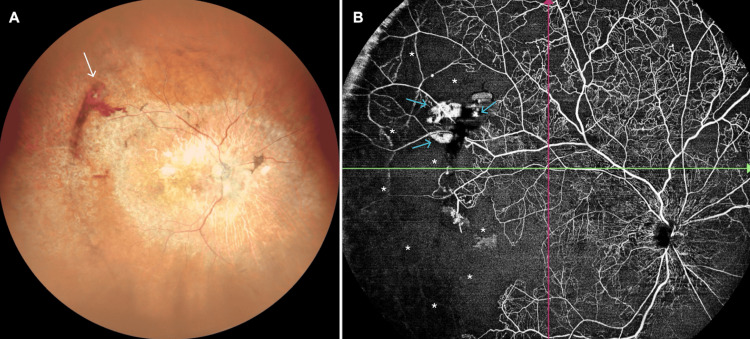
Right eye, 2026 Wide-angle color fundus picture (NIDEK Mirante; Nidek Co., Ltd., Gamagori, Japan) showing the preretinal hemorrhage (white arrow) and laser photocoagulation scars at the superotemporal quadrant (A) and wide-angle optical coherence tomography angiography (BM-400K BMizar; TowardPi Medical Technology Co., Ltd., Beijing, China) showing the nonperfused areas (asterisks) extending almost 270 degrees with retinal neovascularizations (blue arrows) (B).

One month later, the treated areas appeared to be relatively stable (NIDEK Mirante; Nidek Co., Ltd., Gamagori, Japan) (Figure 3A). However, the ischemic regions were observable via wide-angle OCT angiography (BM-400K BMizar; TowardPi Medical Technology Co., Ltd., Beijing, China) (Figure 3B).

Genetic analysis

Total genomic DNA was extracted from 4 mL of the patient's peripheral blood using the Lab-Aid 824s DNA Extraction Kit (Zeesan, China) according to the manufacturer’s procedures. DNA quality and concentration measurements were performed by NanoDrop ND1000 Spectrophotometer (Thermo Fisher Scientific, Waltham, MA). After proper quality (50-100 ng/µl concentration and A260/A280: 1.8-2.0 purity) of DNA was ensured, the DNA was stored at -20°C until further use. Thereafter, clinical exome sequencing (CES) was performed using the Watchmaker DNA Library Prep kit (Watchmaker Genomics, Boulder, CO) according to the manufacturer’s protocols. The library was sequenced paired-end on the NovaSeq platform (Illumina, San Diego, CA). A read depth of 20× for >99% of the exons and exon-intron boundaries of the CYP4V2 gene (transcript no: NM_207352.4) was achieved. Secondary and tertiary analysis of CES data was performed using the SEQ (Genomize, Istanbul, Turkey) web-based platform. Alignment and variant calling were performed against the reference human genome (Hg38). Detected variants were classified according to current guidelines [9]. A homozygous c.254G>A (p.Arg85His) missense variant in exon 2 of the CYP4V2 gene was identified. 

## Discussion

Several macular complications and some rare incidental findings were well-documented in BCD [10]. However, the coexistence of BCD and RVO is extremely rare. In the two largest patient cohorts, one comprising 208 patients [11] and the other comprising 164 patients [12], no cases of RVO were reported. Furthermore, only a single case involving unilateral tributary RVO has been documented in the literature, albeit without any detailed description [7]. On the other hand, Paxhia et al. [13] described a 70-year-old with retinitis pigmentosa and Usher syndrome who experienced unilateral visual deterioration associated with an ischemic type of CRVO, confirmed by fluorescein angiography. Additionally, concurrent macular edema was also observed, but no neovascular complications occurred during the 18-month follow-up period. They hypothesized that VEGF production was not possibly meaningful despite the presence of extensive nonperfused areas, attributable to the thinner retinal vasculature in regions exhibiting bone spicule pigmentation. Following a thorough review of this paper and the existing literature, we did not foresee any form of neovascularization in our case at the time of CRVO detection. Nevertheless, Li et al. [11] categorized BCD into two clinical subtypes: peripheral and central types. They argued that patients with the central type exhibited poorer central vision but demonstrated superior overall retinal health, as observed in our patient. Therefore, extensive ischemic areas in the peripheral retina led to retinal neovascularization as the retinal periphery was relatively intact in the present case. 

Since the central vision was already impaired, the current patient did not report further visual disturbance despite the occurrence of CRVO and the evolution of preretinal hemorrhage afterward. Thus, the presence of RVO and the occurrence of retinal neovascularizations could only be detected during the routine examinations conducted four years apart. Our experience points out the significance of longitudinal follow-up with multimodal imaging in patients with BCD. Systemic risk factors should also be managed proactively to prevent potential vascular complications. 

## Conclusions

The present case highlights a rare coexistence of BCD and CRVO, demonstrating that important retinal vascular complications may occur even in the absence of new visual symptoms. The development of peripheral ischemia and subsequent retinal neovascularization suggested that relatively preserved peripheral retinal integrity in the central type of BCD might have resulted in increased VEGF levels in our patient. This contrasts with previous thinking that neovascular complications are unlikely in dystrophic retinas with advanced chorioretinal degeneration. Our findings emphasize that routine eye examinations may detect additional asymptomatic fundus changes in a timely manner and prevent vision-threatening complications in such rare cases.
